# Did the general practice residents well adapt to real public health prevention ——a study from the COVID-19 prevention training in China

**DOI:** 10.1186/s12909-022-03882-x

**Published:** 2022-12-01

**Authors:** Xin Rao, Li Luo, Qiaoli Su, Xingyue Wang

**Affiliations:** 1 Department of General Practice, General Practice Center, West China Hospital of Sichuan University, Chengdu, China; 2 Institute of Hospital Management, West China Hospital, SCU, Chengdu, China; 3 Sichuan University, China, Chengdu, China; 4 Department of Graduate Medical Education, West China Hospital of Sichuan University, China, Chengdu, China

**Keywords:** GP resident, GP training, COVID-19, Community prevention

## Abstract

**Object:**

GP residents’s has the obligation to take task with the public health prevetion. GP residents receive the public health training during their college study period and the residents training.

The sudden outbreak of the COVID-19 epidemic, highlight the importance and competence of the community prevention as the front line of epidemic prevention and control, pushing the general practitioner (GP) residents into the front team of epidemic prevention and control. Residents’ participation in epidemic prevention and control is not only a field workload participation in public health disease prevention and control, but also a rare and value-oriented training experience. This study aims to explores the research on the training content, ability improvement and cognitive load of the resident, and to demonstrate past and future training effects of epidemic prevention and control.

**Methods:**

Object cognitive load scale (NASA-TLX scale) and self-developed questionnaires were adopted to conduct a questionnaire survey on resident doctors who were in GP training program from West China Hospital of Sichuan University, and finally 190 questionnaires were collected. SPSS 23.0 statistical software for statistical analysis of data.

**Result:**

Most indicators of cognitive load NASA scale are at a “moderate” level by the GP residents, generally indicating that the intensity of on-site epidemic prevention and control (training) can be tolerated. The chi-square test is used to study the status of “How responsible you are for epidemic prevention and control in a community in the future”, the residents grade shows no significant difference while “ how many months after the outbreak when you participated in the epidemic prevention” shows significant difference, the result show that GP residents already have konwledge and skills for the public health prevetion, they need more attitude and mental preparation. Continuing education will have a significant positive relationship with the GP residents’s confidence of the prevetion ofpublic health prevetion.

**Conclusion:**

Former medical school education and training at the regulatory training stage have a good effect for residents to master the ability of epidemic prevention and control, and to prepare for the needs of epidemic prevention and control physically and mentally. After this stage, the epidemic prevention and control training under the real situation will make a great contribution to the self-assessment and performance improvement of the final general practitioners.

**Supplementary Information:**

The online version contains supplementary material available at 10.1186/s12909-022-03882-x.

## Background

The sudden outbreak of the COVID-19 epidemic, highlight the importance and competence of the community prevention. Before the outbreak of the COVID-19 epidemic, medical student and GP residents have receive relevent public health prevention konwledge and sill in college and hospital [1, 2]. To improve emergency response capabilities of public health professionals in significant public health events, some scene simulation teaching in significant public health events including investigation and management training such as situational settings, desktop deduction, situational deduction, self evaluation and teacher summary [3]. But how the training go on, there is no other real site than the real COVID-19 epidemic to exam the GP residents gainning [4, 5] This study aims to explores the research on the training content, ability improvement and cognitive load of the resident at different time points (before, in and after the outbreak of the epidemic) in a real situation, to used in the study of the resident training process, and to demonstrate past and future training effects of epidemic prevention and control.

### Taining content

All participants are rotating general practitioners from West China Hospital of Sichuan University. Whether to participate in the epidemic prevention work, the original training plan shall prevail (whether to rotate the community internship base). According to the needs of prevention and control work, West China Hospital of Sichuan University arranged trainees to rotate to various epidemic prevention and control posts in community health centers to effectively participate in the prevention and control work, and assigned special personnel to be responsible for teaching and support. Under the guidance of superior physicians, residents gradually became familiar with the working procedures. 1. Pre-examination and triage: participate in pre-examination and triage, be familiar with the working process and norms, master the key points of pre-examination (temperature measurement, epidemiological history collection, simple query of clinical symptoms, and make full use of general practice thinking), and be familiar with the triage process (outpatient clinic, general outpatient clinic, fever clinic and referral). 2. Medical observation of quarantined persons 3. Participate in the epidemic prevention and control and daily work of the family doctor team.

## Method

The NASA Mission Load Index (TLX) is a popular technique for measuring subjective mental load [6–8]. It relies on a multidimensional structure to derive an overall workload score based on a weighted average of scores on six subscales: using the Object Cognitive Load Scale (NASA-TLX Scale) and a self-research questionnaire, A total of 190 questionnaires were collected by using the Delphi method to conduct a questionnaire survey on residents who are training general practitioners in West China Hospital of Sichuan University. Questions 8–12 are from the NASA-TLX Scale Questionnaire. Other questions were conducted using the Delphi method based on training needs. A total of 20 preliminary surveys were conducted for this survey, and all of them were sent out after the preliminary survey was revised. Electronic receipts are made through the “Questionnaire Online” APP, and the receipt rate is 100%. Finally, the NASA-TLX scale and the self-developed questionnaire were combined into one questionnaire [9].

## Result

Through pre-survey and statistic analyze, the Cronbach’s alpha coefficient of the questionnaire is 0.91.Spearman-Brown coefficient is 0.98，the survey’sreliability is good .

Comparing the chart above, most indicators of cognitive load [10–13] NASA scale are at a “moderate” level by the GP residents, generally indicating that the intensity of on-site epidemic prevention and control (training) can be tolerated. However, there are still some who are not in a good state. The real epidemic prevention work is a challenge for every GP residents. Although some are in a state of stress, they all successfully completed the epidemic prevention training.

Multi-class Logit regression analysis is use. “How responsible you are for epidemic prevention and control in a community in the future” is as a dependent variable, the independent variableare: 1. Have you participated in the community epidemic prevention work? 2 What is your status when you participated in the epidemic prevention work? 3 You participated in the epidemic prevention and control a few months after the outbreak. The epidemic prevention training you received before came from: Undergraduate Education, Regulatory Training Stage, continuing education, online self-study [14–17] or other, pre-employment training for epidemic prevention comes from: undergraduate teachers, teachers from relevant departments of the hospital, teachers from community bases, grass-roots governments, street offices and hotel staff. The result shows continuing education will have a significant positive relationship with the level of Y and the odds ratio (OR value) is 4.475 (when changing from under the guidance of the teacher to being able to complete it independently). Continuing education will be put highlight of the public health prevetion training in the future. The result also shows the full coverage of training in undergraduate education, resident training, continuing education, online self-study or others (Table 1).

**Table 1:** Cross (Chi-square) analysis results to analysis the influence of the resident grade

What level can you achieve If you are responsible for epidemic prevention and control in a community in the future	Residents grade	Under the guidance of the supervision	Participants can accomplish it independently	Good: participants can instruct junior or other students	Participants have more constructive suggestions	Not sure	Sum	χ^2^	*p*
	not participate in	30(31.58)	6(12.77)	5(22.73)	2(22.22)	6(35.29)	49(25.79)	21.377	0.164
	grade 1	29(30.53)	11(23.40)	3(13.64)	2(22.22)	5(29.41)	50(26.32)		
	grade 2	24(25.26)	17(36.17)	6(27.27)	3(33.33)	3(17.65)	53(27.89)		
	grade 3	11(11.58)	10(21.28)	5(22.73)	1(11.11)	3(17.65)	30(15.79)		
	grade above	1(1.05)	3(6.38)	3(13.64)	1(11.11)	0(0.00)	8(4.21)		
total		95	47	22	9	17	190		

The chi-square test (cross analysis) is used to study the status of “ how many months after the outbreak when you participated in the epidemic prevention “ for “if you are responsible for the epidemic prevention and control work of a community in the future, can you The level achieved”‘show a significant difference. This shows that the GP resident has prepared sufficient knowledge and skills in the epidemic prevention work (Table 2).

**Table 2:** Cross (Chi-square) Analysis results table 2 analysis the influence of the outbreak time of the pandemic

What level can you achieve If you are responsible for epidemic prevention and control in a community in the future	Residents grade	Under the guidance of the supervision	Participants can accomplish it independently	Good: participants can instruct junior or other students	Participants have more constructive suggestions	Not sure	Sum	χ^2^	*p*
	not participate in	30(31.58)	6(12.77)	5(22.73)	2(22.22)	6(35.29)	49(25.79)	21.377	0.164
	grade 1	29(30.53)	11(23.40)	3(13.64)	2(22.22)	5(29.41)	50(26.32)		
	grade 2	24(25.26)	17(36.17)	6(27.27)	3(33.33)	3(17.65)	53(27.89)		
	grade 3	11(11.58)	10(21.28)	5(22.73)	1(11.11)	3(17.65)	30(15.79)		
	grade above	1(1.05)	3(6.38)	3(13.64)	1(11.11)	0(0.00)	8(4.21)		
total		95	47	22	9	17	190		

The chi-square test (cross analysis) is used to study the status of “you participated in the epidemic prevention work (at which year during the residents training period)” for “if you are responsible for the epidemic prevention and control work of a community in the future, can you The level achieved”‘does not show a significant difference. This shows that the GP resident has prepared sufficient knowledge and skills in the epidemic prevention work.

## Discussion

How to enhance public health service utilization in public health, community prevention and how to train our GP residents to make them full cultivated for the public health prevention is a new key issue we confront [18–20]. The sudden outbreak of the COVID-19 epidemic, highlight the importance and competence of the community prevention as the front line of epidemic prevention and control, pushing the GP residents into the front team of epidemic prevention and control [21, 22]. Residents’ participation in epidemic prevention and control is not only a field workload [23, 24] participation in public health disease prevention and control, but also a rare and value-oriented training experience.. The GP residents need to make themselves fully ready in public health knowledge, skill and attitude construction.

### Advantage of the study

The sudden outbreak of the COVID-19 epidemic is rare in history [25, 26], but it leaves the study a unique case and treasure study material.

Object cognitive load scale (NASA-TLX scale) and other method are applied to measure the public health training outcome, especially implicate it for the past, right time and future.

### Limitation of the study

The health personnel training mechanism for responding to public health emergencies still continuous improvement and follow-up research, especially the RDD method to reveal the effect of public health prevention training at different stage of the COVID-19 pandemic.

## Supplementary Information

Additional file 1. Result of the restropective  survery of GP training for prevetion during the COVID-19.

## Data Availability

All data generated or analysed during this study are included in this published article and its supplementary information files.
